# On reactive oxygen species measurement in living systems

**Published:** 2015

**Authors:** LA Pavelescu

**Affiliations:** * Division of Cellular and Molecular Medicine – Human Developmental Medicine, Department of Morphological Sciences, “Carol Davila” University of Medicine and Pharmacy, Bucharest, Romania

**Keywords:** ROS, 2’-7’ Dichlorofluorescin diacetate, RNS, RCS, free radicals

## Abstract

Studies devoted to the detection and measurement of free radicals in biological systems generally generated accepted methods of reactive oxygen species (ROS) level analysis. When out of control, ROS induces tissue damage, chronic inflammatory processes and cellular functional disturbances. Aerobic organisms have adapted to defense against ROS aggression by developing potent antioxidant mechanisms. Recent advances in ROS measurement methodology allow the study of ROS biology at a previously unachievable level of precision. However, their high activity, very short life span and extremely low concentration, make ROS measurement a challenging subject for researchers.

## Introduction

It is well known that the exposition to certain noxious factors, such as infectious agents, pollution, UV light, radiation and cigarette smoke, may lead to the production of ROS [**34**,**35**].

ROS are any oxygen-containing compounds that are particularly reactive. ROS are present in all aerobic organisms, which have evolved defense against their potentially damaging effects as well as ways to utilize them. Some ROS are free radicalsin which an atom has one or more unpaired electrons in its outer orbital, making it particularly reactive. The best-known free radicals in the body are oxygen-based (although other atoms can also exist as free radicals) and are generated as by-products of oxidative metabolism.

The term free radical often used in biology refers to a variety of highly reactive molecules that can be divided into several categories:

 -reactive oxygen species (ROS);

-reactive nitrogen species (RNS);

-reactive chlorine species (RCS).

The most prominent members in the mentioned groups are: Superoxide Anion (O2.-), Hydroxyl Radical (HO.), Peroxyl Radical (ROO.),Hydroperoxyl radical (HO2.), Hydrogen Peroxide (H2O2), Hypochlorous Acid (HOCl), Ozone (O3), Singlet Oxygen(1O2),Nitric Oxide (NO.), Nitrogen Dioxide Radical (NO2.), Nitrous Acid (HNO2), Nitrosyl Cation (NO+), Nitrosyl Anion (NO-), Atomic Chlorine (Cl-), Hypochlorous Acid (HOCl), Chorine (Cl2), Nitronium Chloride (NO2Cl) in reactive chlorine species. These various radical species can be generated exogenously or produced by different sources inside the cells [**1**-**4**].

On the other hand, ROS as well as RNS and non-radicals, like peroxynitriteanion (ONOO-), peroxynitrous acid (ONOOH), nitrosoperoxycarbonate anion (ONOOCO2-), nitronium cation (NO2+), dinitrogen trioxide (N2O3) and dinitrogentetraoxide (N2O4) are continuously generated in smallquantities during normal cellular processes [**36**].

It is known that free radicals are essential in tissue defense mechanisms, which are accomplished by neutrophils, macrophages and other cells,which are part of the immune system. They are very important in the molecular processes of cells, but their overproduction may lead to cell and tissue irreversible damage. To avoid an excess of free radicals,the cell antioxidants are trying to keep their production and consumption balance [**5**-**7**].

Endogenously produced reactive oxygen species are essential tolife, being involved in different biological functions, namely neurotransmission, peristalsis, platelet aggregation, blood pressure modulation, smooth muscle relaxation, immune system control, phagocytosis, the cellgrowth regulationand cellular signaling.

**Fig. 1:**
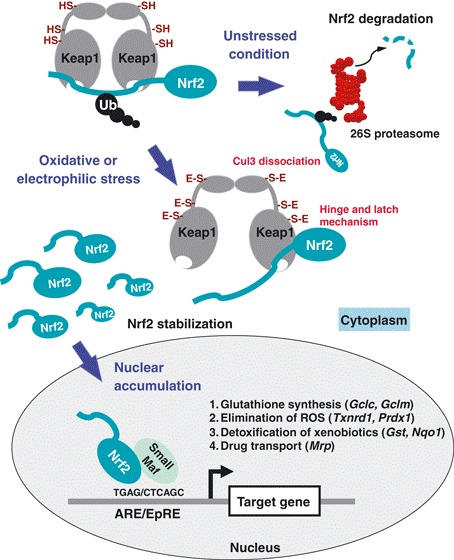
Molecular mechanisms of the Keap1–Nrf2 pathway in stress response and cancer evolution [**37**]

This is an example of how ROS may be involved in intracellular signaling events. The transcription factor Nrf 2 plays an important role in glutation synthesis, elimination of ROS, detoxification of xenobiotics and drug transport. Keap 1 (Kelch-like ECH-associated protein 1) is a cytoplasmic protein essential for the Nrf 2 regulation activity. The regulation of Nrf 2 levels by Keap 1 is not functional in some human cancer types, when the interaction between Nrf 2 and Keap 1 is broken; due to this fact, the Nrf 2 activation contributes to drug resistance by efflux of cancer drugs as well as to cancer cells proliferation.

## Detection of reactive oxygen species

Spectrophotometry and chemiluminescence are example of very used methods in the detection of reactive oxygen species. 

Products resulting from DNA damage by free radicals are determined in urine as 8-hydroxydeoxyguanosine (8-OHdG), which is the most widely determined compound.Apart from this, one can determine compounds such as 8-hydroxyadenine and7-methyl-8-hidroxyguanine [**9**].

Highlighting lipid peroxidation is important in many diseases; it makes, for instance, a fundamental contribution to atherosclerosis [**10**]. Many assaysare available to measure lipid peroxidation, such asmalonic dialdehyde (MDA) by the thiobarbituric acid (TBA) test and diene conjugation.Recently, ferrous oxidation with Xylenol Orange (FOX) assay coupled with triphenylphosphine has shown to be a reliable marker in determining levels of hydroperoxides(ROOH).

8 epi-prostaglandin-F2α (8-epi-PGF2α) is a marker of oxidative stress derived from oxidation of phospholipids containing arachidonic acid.

Oxidative damage to proteins is important as it influences the function of receptors, enzymes and transport proteins. -SH oxidation, carbonyls, aldehyde adducts oxidized tyr, trp, his, met, lys, leu, ileu, val, protein peroxides/hydroxides are specific markers which show that oxidative stress has occurred [**9**-**13**].

Experimental determination of the ROS production can be done by various methods with different specificity and sensitivity; the free radicals may be determined as particular species or as a global amount of oxidative species. The most widely used methods are those based on the interaction of chemical compounds with reactive species, which leads to a consecutive fluorescence emission or chemiluminescence. Today, the commonly used fluorescence markers are based on the structure of 2'-7' dichlorfluorescein (DCF) [**31**,**36**]. The probes are acetylated for an easy penetration through the cell membrane. Cytoplasmic esterases cleave the acetyl group, releasing the reduced state of DCF (DCF-H2) which can then react with intracellular reactive species. DCF-H2 is retained in the cell where it interacts with the reactive species and fluoresces upon oxidation.

**Fig. 2:**
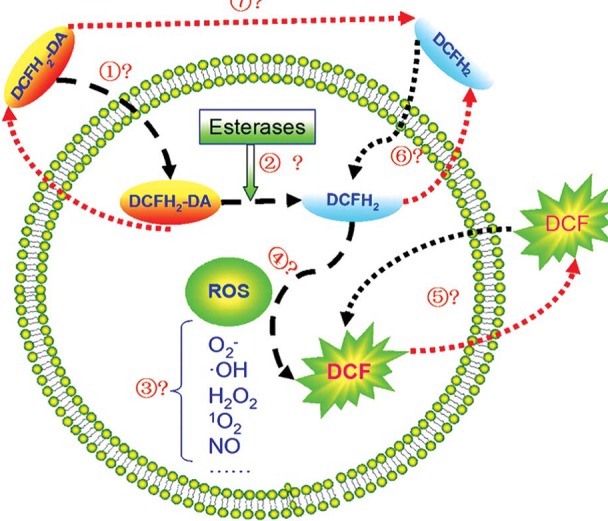
The mechanism of DCFH2-DA/ DCFH2 action in cells

DCFH2-DA is a non-fluorescent lipophilic probe that can cross the cell membrane. Inside the cell, DCFH2-DA deacetylates to form DCFH2, which is also a non-fluorescent probe but cannot diffuse freely across the cell membrane. DCFH2 reacts with intracellular ROS to give the fluorescent DCF.

The disadvantage of using these compounds lies in their sensitivity to photo-oxidation and chemical environment. For this reason, it is necessary to use suitable working conditions: low light environment, opaque containers or constant ambient temperature [**14**]. Dichlorodihydrofluoresceindiacetate (DCFH-DA) is the most widely used probe for the detection of intracellular H2O2 and oxidative stress. There are several known limitations and artifacts associated to intracellular H2O2determinations that must be kept in mind when DCFH-DA is used to measure H2O2. DCFH2 does not react directly with H2O2 to form the fluorescent product (DCF), this being the reason why H2O2 is not direct measured with DCF fluorescence. Initially, an intermediate radical DCF- is formed, which in turnreacts with O2 to form superoxide (O2.-), leading to artifactual amplification of the fluorescence signal intensity. Cytochrome c, which is released from mitochondria to the cytosol during apoptosis, canoxidize DCF-H2 to DCF leading to artifactual amplification of the fluorescence signal intensity [**15**-**17**]. 

Other probes for the measurement of intracellular H2O2 arearomatic boronates (ester and boronic acid) that react with H2O2 to form a single major product, the corresponding phenol [**17**-**19**]. Another probe very frequently used to measure H2O2 is Amplex red. Resorufin is the fluorescent product obtained with the help of horseradish peroxidase that oxidizesAmplex red to resorufin. This process is highly efficient in the presence of H2O2 and vastly increases the yield of resorufin [**20**-**22**].

Hydroethidine (HE) is another widely used probe for the detection of intracellular O2.-. The product 2-hydroethidiume (2-OH-E+) with similar fluorescence characteristics is formed from the direct reaction between O2.- and the HE [**23**-**27**]. The mitochondrial O2.- is measured with mitochondrial-targeted HE or Mito-SOX, a triphenylphosphoniumcation conjugated to HE. Mito-SOX reacts with O2.-and forms a red fluorescent product, 2-hydroxymitoethidium (2-OH-Mito-E+) [**29**-**30**]. For the measurement of ONOO-the most used probe is dihydrorhodamine (DHR). The oxidative mechanism of DHR is similarto DCFH oxidation in many aspects. DHR is oxidized to a fluorescent product named rhodamine. The DHR oxidation is not induced directly by ONOO-, being mediated by oxidants like NO- and OH. [**30**].

The reactive species formed in biological systems should be detected by using different methods. Myhrea et al. suggested that DCFH is sensitive toward oxidation by ONOO-, OH., H2O2 in combination with cellular peroxidases. H2O2 alone does not oxidize DCFH into DCF and this is why it is not commonly used for the measurement of NO, HOClor O2.-, in biological systems. The DCF formation is rapidly increasing due to small quantities of these oxidants, while other oxidants may need higher concentrations and longer incubation time [**31**, **8**].

Due to their specificity, chemiluminescent probes, luminol and lucigenin are frequently used in ROS evaluation studies. The lucigenin and luminol probes are made chemiluminescent by O2- and HOCl, but are not suitable for the detection of NO, ONOO-, H2O2 or OH.inliving systems. Lucigenin appears rather specific for O2.-but not for H2O2 or for HOCl [**31**]. The validity of lucigenin as a chemiluminescence probe has been questioned due to the fact that it may itself act as a source of O2.-[**32**,**33**], in particular in cellular systems, in which there is a significant production of O2.-. In cellular systems, ONOO- induceslucigenin-amplified chemiluminescence [**31**].

A summary of the ROS detection methods, excitation/emission wavelengths, reactant induced fluorescence changes and themain probes used in various applications, can be found in the review “Fluorescence probes used for detection of reactive oxygen species”, by Ana Gomes et al. (2005).

## Conclusion

DCFH2 -DA/DCFH2 are useful probes for ROS production study in cell free and in biological systems; they are more suitable to point out the total ROS production rather than as probes for a specific kind of ROS. As Tarpey and Fridovich (2001) remarked “because of the multiple pathway that can lead to DCF fluorescence and inherent uncertainty relating to endogenous versus artifactual oxidant generation,this assay may best be applied as a qualitative marker of cellular oxidant stress, rather than a precise indicator of rates of H2O2 formation”.
